# Adrenal Mass in a 70-Year-Old Woman

**DOI:** 10.1155/2022/2736199

**Published:** 2022-07-12

**Authors:** Kiana Karimi, Mohsen Nikzad, Sohrab Kulivand, Shiva Borzouei

**Affiliations:** ^1^Student Research Committee, Hamadan University of Medical Sciences, Hamadan, Iran; ^2^Universal Scientific Education and Research Network (USERN), Tehran, Iran; ^3^Department of Internal Medicine, School of Medicine, Hamadan University of Medical Sciences, Hamadan, Iran; ^4^Department of Radiology, School of Medicine, Hamadan University of Medical Sciences, Hamadan, Iran; ^5^Department of Endocrinology, School of Medicine, Hamadan University of Medical Sciences, Hamadan, Iran

## Abstract

**Introduction:**

Adrenocortical carcinoma is a rare endocrine malignancy with a bimodal age distribution pattern that affects women more than men. More than half of the patients present with hormone excess manifestations such as Cushing's syndrome and virilization. Non-functional tumors usually are diagnosed incidentally following imaging studies due to a mass effect or metastatic disease. Surgical resection is considered the best curative treatment for these tumors. *Case Presentation*. A 70-year-old woman presented with a 3-month history of diffuse intermittent abdominal discomfort, weight loss, and additional hair growth. Imaging investigations revealed a large 187 × 85 × 140 mm mass between the liver and upper pole of the right kidney which has displaced the adjacent structures. Hormonal evaluations detected high levels of cortisol and adrenal androgens. She underwent open adrenalectomy and right nephrectomy due to severe adhesion of the mass. Histopathological evaluations revealed adrenocortical carcinoma and the patient received adjuvant radiotherapy.

**Conclusion:**

Precise physical examination, hormonal evaluation, and imaging studies play a key role in differentiating malignant adrenal masses in all patients, especially in those with vague symptoms. Radical excision of the mass and appropriate adjuvant chemotherapy or radiotherapy improve the outcome for patients.

## 1. Introduction

Adrenocortical carcinoma (ACC) is a rare endocrine malignancy with an approximate prevalence of 1 to 2 per million. Although this malignancy can develop at any age, it has a bimodal age distribution pattern which commonly affects patients under the age of 5 or in the 4^th^ to 5^th^ decades of life [1]. In addition to genetic predisposition, there is no definite and known risk factor for ACC. However, some studies have shown smoking in men and the use of contraceptives in women as probable risk factors for ACC [2].

Most patients present with signs and symptoms of hormone excess, especially hypercortisolism (50–80%). About one-third of patients have clinical manifestations of local tumor growth, and 20–30% of them are diagnosed incidentally following imaging studies for other reasons [3]. The initial diagnosis of ACC is based on a careful history and physical examination, along with biochemical and endocrinological evaluations. Computed tomography (CT) scanning and magnetic resonance imaging (MRI) are choice imaging modalities for distinguishing ACCs from adrenocortical adenomas, determining local invasion, and distant metastases [4].

In the pathological evaluation of adrenal tumors, size is an important and determining factor in the diagnosis of ACCs [3]. These tumors are typically large, more than 6 cm and diameters of 4–25 cm [5, 6]. Clinical, histological, and immunohistochemical parameters are also conductive in confirming the diagnosis. The Weiss system is the most acceptable histopathological system for assessing malignancy in adrenocortical tumors [7].

Despite the limitation of knowledge about the treatment and prognosis of these patients due to its rarity, some progress has been made in this regard. Surgical resection of the tumor and suspicious lymph nodes is considered a potentially curative treatment. Adjuvant mitotane therapy is recommended for all patients who are at risk for recurrence or have high-grade or incompletely resected tumors. The addition of postoperative radiation therapy (RT) is also suggested for this group of patients [8].

Herein, we present the case of a 70-year-old woman with abdominal discomfort, weight loss, and terminal hair overgrowth that was diagnosed with adrenocortical carcinoma.

## 2. Case Presentation

A 70-year-old woman, a retired teacher, presented with a 3-month history of abdominal pain. The abdominal discomfort has been intermittent and diffuse without any specific pattern. She has lost her appetite and her body weight decreased about 10 kg over this period. She has complained of malaise and occasional headaches. She denied nausea or vomiting, diaphoresis, palpitation, sleep disturbance, and abnormal uterine bleeding. She has also noted some additional hair growth on her chin, between the breasts, and on the lower abdomen. In her previous medical history, she had controlled hypertension and was on losartan, and she had no history of diabetes. She has also been hospitalized due to COVID-19 one month previously.

On physical examination, she looked cachectic. Her blood pressure was 160/80 mmHg, and other vital signs were normal. On abdominal examination, she had no striae or central obesity. There was mild tenderness over the right upper quadrant and a non-pulsatile 15 cm mass was palpated under the liver. She had excessive terminal hair with a modified Ferriman–Gallwey score of 16. Lower extremities examination showed proximal myopathy in the pelvic girdle. Examination of the cardiovascular and respiratory systems was normal.

**Figure 1:**
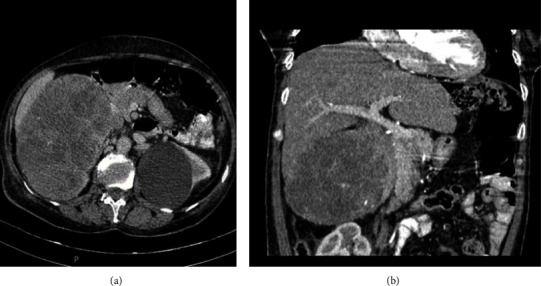
Abdominal CT scan with IV contrast. (a) Axial view of the heterogenous well-defined mass above the right kidney. (b) Coronal view of the adrenal mass effect on the IVC and pancreatic tail.

The initial laboratory tests revealed an elevation in white blood cell count, fasting glucose concentration, and lactate dehydrogenase level. Other findings were all within the normal limits (Table 1). Contrast-enhanced CT of abdomen indicated a solid retroperitoneal 187 × 85 × 140 mm mass with regular borders and heterogenous enhancement located between the upper pole of the right kidney and VI and VII segments of the liver which has pushed the right kidney down (Figure 1). According to these findings, more hormonal evaluations were performed with the abnormal results shown in Table 2. There was no evidence of bone involvement in the bone scan. Findings suggested adrenal carcinoma, and the patient underwent an exploratory laparotomy after controlling hypertension.

**Figure 2:**
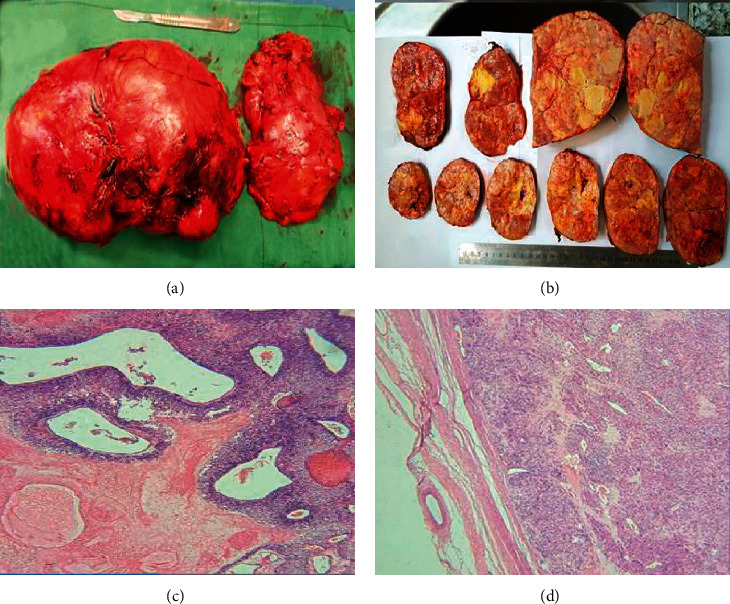
(a) Macroscopical view of the adrenal mass and right kidney. (b) The adrenal mass with a nodular and extensive necrotic area at sectioning. (c) High mitotic activity with multifocal necrosis. (d) Extension of the neoplastic cells to the capsule.

Laparotomy was performed, and the right adrenal mass and kidney were excised due to severe adhesion of the mass to the right renal vein. The inferior vena cava has also been repaired. Macroscopically, the adrenal removed specimen measured 23 × 15 × 9 cm and weighted 1667 g. The tumor was solid, yellowish-gray, and well-defined with an intact capsule. Cross section of the tumor revealed a nonhomogenous nodular area with extensive necrosis. There was no evidence of neoplastic invasion to the capsule or external surface of the mass. Microscopic examination revealed polyhedral neoplastic cells in trabecular structures which were arranged in dense cellular sheets. Tumoral cells had giant and pleomorphic nuclei, vesicular chromatin, marked eosinophilic nucleoli, and large amounts of eosinophilic granular to clear cytoplasm. The mitotic activity was more than 6 in 50/high-power fields (HPF), and multifocal coagulation tumoral necrosis was observed. The capsular invasion was identified, but it was not extended beyond it. There was no evidence of tumoral involvement in the right kidney (Figure 2).

The patient was observed in the intensive care unit for a few days after surgery. She was referred to the oncology ward with no surgical complications 9 days after surgery. Since the mass was completely resected and there was no evidence of distant spread of the tumor, she underwent adjuvant radiation therapy without chemotherapy. Follow-up thoracic, abdominal, and pelvic CT 3 months after surgery revealed no recurrence or metastasis, and all hormonal and biochemistry evaluations were within normal limits. 5 months after surgery, she is alive and back to normal in good condition.

## 3. Discussion

Adrenocortical carcinoma is a rare malignancy in the endocrine system, usually with a poor prognosis [10]. The incidence of these tumors is about 0.7–2.0 cases per million each year, and they are more found in women in the fifth decade of life [11, 12]. They can also affect children, mostly as functional tumors [13]. Most ACC cases appear to be sporadic as in the present case. However, some cases were considered to be associated with several hereditary tumor disorders such as Li–Fraumeni syndrome, multiple endocrine neoplasia type 1, and Beckwith–Wiedemann syndrome [14].

Studies have indicated that more than 60% of ACCs are hormone-producing tumors and they can develop clinical syndromes of hormone excess. These tumors usually induce Cushing's syndrome (45%) in adults and are associated with glucocorticoid excess symptoms, such as weakness, and weight gain, which commonly develop over three to six months. About 25% of patients present with a mixed Cushing's and virilization syndrome, due to excessive secretion of both androgens and glucocorticoids and only fewer than 10% of them induce virilization symptoms, implying ACC rather than adenoma [6, 15]. Our patient's main complaint was abdominal discomfort besides recent weight loss which is related to local mass effect. She had no clinical manifestations of Cushing's or virilization syndrome which may be due to the short period from the onset of the tumor growth.

Hormonal estimation including adrenal androgens and glucocorticoids levels may help to determine the adrenal source of the patient's signs and symptoms, providing tumor markers which are useful during patient's follow-up to assess possible tumor recurrence or residual tumor after surgery [8]. In the present case, high adrenal androgens and estradiol levels were detected and made the diagnosis of adrenal carcinomas more probable than adrenal adenomas [16]. Urinary excretion of metanephrines and normetanephrines was measured, and they were within normal range which excluded the possibility of pheochromocytoma.

CT scan and MRI are preferred imaging methods for evaluating the tumor size, local invasion, or distant tumor invasion. These tumors usually have the following features on CT scan: tumor size larger than 4 cm, inhomogeneous but well-defined suprarenal mass, displacing adjacent structure, heterogenous enhancement after IV contrast administration, and less frequent calcification and IVC tumor thrombus, especially in right-sided tumors [4]. As described above, our patient had most of these features in imaging studies.

Fine-needle aspiration biopsy is not usually recommended for distinguishing adrenal carcinoma from a benign adrenal mass. However, it could be performed when there is a high suspicion of distant metastasis in adrenal glands [17]. The Weiss histopathologic system is commonly used for evaluating the probability of malignancy in adrenocortical tumors. The modified Weiss system has the following criteria: abnormal mitoses, >6 mitoses/50 HPF, ≤25% clear tumor cells in the cytoplasm, necrosis, and capsular invasion [7]. The present case had all the features, which led to establishing the diagnosis of malignancy.

Complete surgical resection is the only potentially curative treatment for stage I to III ACCs (Table 3). However, due to the presence of occult micrometastases, it may not be curative for many patients [15]. Adjuvant mitotane therapy is recommended for all the patients at high risk of disease recurrence after surgery, including all cases with high-grade disease, intraoperative tumor spillage or fracture, incompletely resected tumor, and vascular or capsular invasion [8, 18]. Previously, ACC was considered to be a relatively radioresistant tumor. However, some studies indicated a benefit in those who are at risk of local recurrence [19]. In the present case, the tumor was in stage III and we decided to perform open surgery rather than laparoscopic resection due to the tumor size, uncertainty about the tumor local invasion, and reducing the risk of spillage or fracture. The mass was successfully resected; after histopathologic evaluation, no invasion beyond the capsule was detected, so we preferred radiotherapy to chemotherapy as adjuvant therapy after surgery.

In conclusion, ACC as a rare neoplasm is a great challenge for diagnosis and treatment. Although most adult patients have functional tumors, precise physical examination, hormonal evaluation, and imaging studies should be conducted for all suspicious patients to diagnose both functional and non-functional tumors and differentiate them from adrenal adenomas. Despite the lack of information about a certain treatment, surgical resection is recommended as a potentially curative treatment. Adjuvant chemotherapy or radiotherapy should be considered based on staging and histopathologic evaluation after surgery.

## Figures and Tables

**Table 1:** Initial laboratory workup [9].

Test	Result	Normal range
WBC (white blood cells)	15740/*µ*l	4000–11000
RBC (red blood cells)	5.9 million/*µ*l	4.2–5.4
Hb (hemoglobin)	15.2 g/dl	12.5–16
Platelets	194000/*µ*l	140000–450000
FPG (fasting plasma glucose)	127 mg/dl	60–110
LDH (lactate dehydrogenase)	340 IU/l	140–280
ESR 1h (erythrocyte sedimentation rate)	5 mm/h	0–30
CRP (C-reactive protein)	Negative	
Bun	14 mg/dl	5–23
Cr	1.1 mg/dl	0.6–1.4
Ca	8.6 mg/dl	8.6–11
P	3.4 mg/dl	2.9–5.1
Na	142 mEq/l	135–145
K	3.9 mEq/l	3.9–5.3

**Table 2:** Hormonal and tumor marker evaluation [9].

Test	Result	Normal range
Dehydroepiandrosterone sulfate	>1000 *µ*g/dL	Female: 30–400
Testosterone	8.810 ng/ml	>50y: 0.029–0.408
17-OH-progesterone	25.37 ng/ml	Menopause: 0.2–0.9
Estradiol	76 pg/ml	<60
Androstenedione	4.3 ng/ml	0.9–3.2
Cortisol (8 AM)	46.81 micg/dL	6.4–21
24-hour urinary free cortisol	255.1 *µ*g/24 hrs	1.5–63
24-hour urinary metanephrine	30 *µ*g/24 hrs	<350
24-hour urinary normetanephrine	386.9 *µ*g/24 hrs	<600
AFP (alpha-fetoprotein)	3.74 IU/ml	Up to 5.8
CEA (carcinoembryonic antigen)	1.1 ng/ml	Non-smokers: <5.0
CA 125 (carbohydrate antigen 125)	23.48 U/ml	Up to 35

**Table 3:** Classification system for staging ACC [9, 20].

ENSAT stage	TNM stage	TNM definition
I	T1, N0, M0	T1, tumor ≤5 cm
		N0, no positive lymph node
		M0, no distant metastases

II	T2, N0, M0	T2, tumor >5 cm
		N0, no positive lymph node
		M0, no distant metastases

III	T1-T2, N1, M0	N1, positive lymph node(s)
	T3-T4, N0-N1, M0	M0, no distant metastases
		T3, tumor infiltration into surrounding tissue
		T4, tumor invasion into adjacent organs or venous tumor thrombus in vena cava or renal vein

IV	T1-T4, N0-N1, M1	M1, presence of distant metastases

ENSAT, European Network for the Study of Adrenal Tumors; TNM, tumor, node, metastasis.
